# Girl Champ in eSwatini: A Strategic Marketing Campaign to Promote Demand for Sexual and Reproductive Health Services Among Young Women

**DOI:** 10.1007/s10461-021-03446-y

**Published:** 2021-08-31

**Authors:** Marie A. Brault, Sarah Christie, Amanda Manchia, Khabonina Mabuza, Muhle Dlamini, Erika L. Linnander

**Affiliations:** 1 Department of Social and Behavioral Sciences, Yale School of Public Health, 60 College Street, New Haven, CT 06510-3201 USA; 2 Department of Health Policy and Management, Yale School of Public Health, New Haven, CT 06510 USA; 3 Global Health Leadership Initiative, Yale School of Public Health, New Haven, CT 06510 USA; 4 School of Public Health, University of the Western Cape, Bellville, South Africa; 5 Project Last Mile, Johannesburg, South Africa; 6 Ministry of Health, Government of the Kingdom of eSwatini, Mbabane, Eswatini

**Keywords:** Adolescents, Sexual health, HIV, Strategic marketing, eSwatini, Mixed methods

## Abstract

Efforts to engage adolescent girls and young women (AGYW) in HIV services have struggled, in part, due to limited awareness of services and stigma. Strategic marketing is a promising approach, but the impact on youth behavior change is unclear. We report findings from a mixed methods evaluation of the Girl Champ campaign, designed to generate demand for sexual and reproductive services among AGYW, and piloted in three clinics in the Manzini region of eSwatini. We analyzed and integrated data from longitudinal, clinic-level databases on health service utilization among AGYW before and after the pilot, qualitative interviews with stakeholders responsible for the implementation of the pilot, and participant feedback surveys from attendees of Girl Champ events. Girl Champ was well received by most stakeholders based on event attendance and participant feedback, and associated with longitudinal improvements in demand for HIV services. Findings can inform future HIV demand creation interventions for youth.

**Supplementary Information:**

The online version contains supplementary material available at 10.1007/s10461-021-03446-y.

## Introduction

In Sub-Saharan Africa, four in five new human immunodeficiency virus (HIV) infections occur among adolescents aged 15–19 years old, with adolescent girls and young women (AGYW) disproportionately affected [1–5]. The Kingdom of eSwatini has the highest rates of HIV in the world, and HIV prevalence is five-times higher among young women 20–24 years old (20.9%) than young men of the same age (4.2%) [6]. Despite their high risk, youth continue to have lower HIV testing and treatment rates, poorer sexual and reproductive health (SRH) outcomes, and are consistently missed by large-scale “treatment as prevention” efforts in Sub-Saharan Africa [7–11]. Efforts to improve the uptake of SRH services globally including pre-exposure prophylaxis (PrEP) and HIV testing and care among adolescent girls and young women have been hindered by fragmented programs and services, poor outreach and communication, and limited service demand [12–19]. Young women describe limited awareness of available services, stigma associated with accessing services and discussing SRH, and negative experiences with healthcare providers as barriers to care [20–23].

Strategic marketing has great potential to engage adolescent girls and improve their uptake of HIV and SRH services [24–26]. Strategic marketing employs private sector marketing principles to drive targeted health communications by segmenting the population and developing and deploying targeted messaging to those subsets of the population [27]. Strategic marketing has been used to develop effective messaging to promote voluntary medical male circumcision [28], adherence to antiretroviral therapy (ART) [29], and uptake of HIV testing among men who have sex with men (MSM) [30, 31], and is increasingly recommended to develop youth-centered branding for effective HIV prevention in the era of PrEP and “test and start” [12, 27, 32]. In particular, strategic marketing can be applied to shift HIV messaging for youth away from risk-oriented or loss-framed approaches towards wellness and gain-framed approaches [33–37]. Despite recent calls to incorporate strategic marketing interventions into HIV programming, there are few studies that evaluate these campaigns in practice or assess their impact on health behavior change [32, 38, 39].

Here, we report findings from the mixed methods evaluation of the Girl Champ campaign in eSwatini, developed through a partnership between the eSwatini Ministry of Health (MoH) and Project Last Mile (PLM), and piloted in three public clinics in the Manzini region [40]. Girl Champ, described in more detail below, was designed to generate demand for SRH services among AGYW through clinic-based events for AGYW and enhanced youth-centered training for clinic staff. Findings from the Girl Champ pilot can inform scale-up of Girl Champ in eSwatini, as well as guide others seeking to incorporate private sector marketing approaches to implement gain-framed HIV prevention interventions for AGYW in low- and middle-income settings.

## Methods

Our mixed-methods evaluation of the Girl Champ pilot integrates data from three sources: longitudinal, clinic-level data on health service utilization among AGYW before and after the pilot; qualitative interviews with stakeholders responsible for the development and implementation of the pilot; and participant feedback surveys from AGYW attendees of Girl Champ events. We integrated data from these three sources to assess the effectiveness of the pilot and to identify facilitators and barriers to implementation [41]. This study was approved by the Yale Institutional Review Board and the eSwatini Ministry of Health.

### Setting

The Kingdom of eSwatini is home to roughly 1.3 million people, and divided into four regions which are further subdivided into 55 *tinkhundla* (municipalities). Over a third of the population is between the ages of 10–24 years old [42]. This project was situated within Manzini region, which had an estimated population of 360,120 in July, 2017 [43]. An estimated 10.5% of Manzini’s population are AGYW between 15 and 24 years of age. Girl Champ was piloted in the *tinkhundla* served by three clinics in the Manzini region: Mafutseni Clinic, Lamvelase Zombodze Clinic, and Luyengo Clinic.

The Ministry of Health (MoH) provides oversight of clinical services, including primary and preventive health services. Preventive health programs include the Swaziland National AIDS Program (SNAP) and the Sexual Reproductive Health (SRH) program which spearheads the youth-friendly service initiative. The MoH also supports the Health Promotion Unit (HPU), responsible for national health communications. The National Emergency Response Council on HIV and AIDS (NERCHA) is a clearinghouse for HIV information and support, which facilitates coordination of national HIV prevention activities as a principal recipient of the Global Fund.

This project was led by Project Last Mile (PLM), and situated within the HPU of the MoH, supported by the Global Fund in partnership with NERCHA. PLM is a global health partnership facilitated between ministries of health and The Coca-Cola Company that aims to integrate best practices from the private sector into public sector agencies across Africa. In eSwatini, PLM applied strategic marketing expertise from Coca-Cola to improve demand for health services in the public health sector. Further details on PLM have been published elsewhere [40, 44, 45].

### Description of the Girl Champ Intervention

PLM worked with local stakeholders to co-create Girl Champ using participatory market research approaches that are typically deployed in the private sector to design communications that attract consumers to brands and products [46–50]. As previously described [51, 52], the in-depth market research findings revealed young emaSwati women aspired to defend themselves against HIV with accurate knowledge, compassionate health care, and community and peer support. They sought safe spaces where they could openly discuss and address their health needs. Clinics were regarded as sources of credible information and quality health services, but AGYW did not visit facilities due to stigma associated with youth sexuality and for fear of judgment from nurses and clinic staff who viewed AGYW as children too young to engage in sexual activity. Building on this market research, Girl Champ was developed by Coca-Cola’s regional creative agency, Foote, Cone & Belding (FCB) Africa, as a patient-centered aspirational brand where young women can access information, support, health services, and wellness activities in a girls-only environment during clinic-based events [52]. An accompanying “Coach” curriculum was developed to enhance training for all clinic staff in youth-responsive care, ensure consistent messaging with AGYW, and overcome some of the stigma and judgement that prevented youth from seeking clinic care. During the Coach training sessions, healthcare workers and staff from participating clinics were refreshed on youth-friendly service delivery by the International Center for AIDS Care and Treatment Programs (ICAP), an NGO providing capacity-building support in the region.

Girl Champ relied on community mobilization to raise awareness, and AGYW were mobilized to attend Girl Champ events (called “activations”) through PLM-facilitated outreach with schools, clinic health committees, and traditional councils that endorsed their attendance. Local textiles were incorporated into the promotional materials disseminated through these outlets. AGYW were provided transportation to attend the day-long events, which were held at three pilot clinics on three consecutive weekends in November, 2018. Events were held in a branded marquee with a boxing ring at the center. The events consisted of live music and entertainment from a local DJ celebrity, fitness activities including boxing demonstrations and yoga, as well as candid testimonials from healthcare workers followed by interactive Q&A. Onsite health services (including HIV testing and counseling) were also offered. Attendees were given merchandise to wear reflecting the Girl Champ brand with a lioness logo inspired by their aspirations to defend themselves against HIV. All attendees at Girl Champ events were registered in the Client Management Information System (CMIS) onsite. This enabled tracking of attendance at all Girl Champ events and identification of AGYW who were newly registered for healthcare services at the events. Written head of household consent, youth proof of age and verbal assent was provided for all AGYW prior to their participation in Girl Champ events.

### Quantitative Data Collection and Analysis

#### Event Reach and Attendance

Event attendance was collected from sign-in and registration forms at each event. To calculate the percentage of the population reached, 2017 Census projections were used [43], where it was projected that AGYW made up 10.5% of the population of participating tinkhundla, based on Manzini census estimates.

#### Health Service Utilization

Health service utilization data were extracted from the CMIS by the Health Management Information System at the MoH. Monthly caseload data were available from July, 2018 through March, 2019 from two sites (Luyengo and Mafutseni). Zombodze maintained paper-based records, which were not available and therefore excluded from this analysis. Health service utilization data (caseloads) and HIV testing and counselling (HTC) visits were stratified by sex and age to examine differences in utilization across subgroup. We assessed average overall health service utilization of AGYW during the 4 months prior to the Girl Champ events and during the 4 months after the events, exclusive of the month when the event was held (November, 2018). Descriptive statistics and chi-square tests were conducted to compare the proportion of health center visits and HTC visits which were utilized by AGYW before and after the Girl Champ events. All statistical analyses were performed in SAS 9.4.

#### Participant Feedback

AGYW who attended Girl Champ events were asked to complete paper-based evaluation forms, which were manually entered into an Excel database. The evaluation forms consisted of a 16-item questionnaire with questions on demographics, where girls learned about the event, what attendees liked or disliked about the event, and suggestions for improvement.

### Qualitative Data Collection and Analysis

As previously described [45], the evaluation of PLM relies on participatory, qualitative research to explore the strengths, challenges and lessons learned from implementation of the partnership. We conducted in-depth, in-person interviews with ‘key informants’ (those with practical experience with the Girl Champ pilot in eSwatini) in March, 2019 [53]. Interviews followed a discussion guide (see Supplemental Materials) with content probes to explore participant perceptions of the pilot intervention; receptivity to the strategic marketing process and the resulting Girl Champ brand; barriers and facilitators to implementation; and sustainability. Interviews lasted 45–60 min. All interviews were audio-taped with participants’ consent and professionally transcribed by an independent transcription service.

We used a purposeful sampling approach with snowball sampling to identify 19 key informants [54], representing a variety of organizations as shown in Table 1. Sampling and interviews continued until thematic saturation was reached.

**Table 1:** Distribution of interview participants by organizational affiliation

Organization	# of participants
Project last mile team	2
Public sector partners^a^	7
Private sector: advertising agency	2
Regional implementing partners, including clinic staff and HMIS/M&E	8
Total	19

^a^Swaziland Ministry of Health and Health Promotion Unit; Swaziland National AIDS Program (SNAP); School Health Program; NERCHA

A four-person multidisciplinary team (including graduate-level anthropology, public health, social work, health services, qualitative methods, HIV, and adolescent health expertise) used the constant comparative method to conduct line-by-line review and coding of the data in Atlas.TI v8. The codebook (see Supplemental Materials) was iteratively developed over five drafts throughout the coding process [54]. Team members were encouraged to challenge discrepant views, and the codebook included codes for disconfirming information. Thematic analyses were conducted through in-depth review of the code reports with reflection on the factors that facilitated and/or hindered implementation of the Girl Champ pilot.

## Results

We begin by describing the effectiveness of the Girl Champ pilot in terms of reach and associated changes in uptake of services. This is followed by qualitative and quantitative findings describing the facilitators and barriers to implementation of the pilot that may have influenced the outcomes.

### Effectiveness of Girl Champ

A total of 1,722 AGYW attended the events (Table 2). Of these, 73.4% (n = 1264) were newly registered for services based on entries into the CMIS system. This integration with the CMIS system was viewed by stakeholders as a useful feature of Girl Champ, offering potential for follow-up with attendees. According to 2017 Census projections [43], the intervention reached approximately ~ 4.6% of young women aged 15–24 years old in Manzini broadly (1722 out of 37,793 AGYW), and 21.1% of those from participating tinkhundla, including 16.1% (n = 570) in Ludzeludze (Zombodze Clinic); 23.5% (n = 544) of AGYW in Mafutseni (Mafutseni Clinic); and 26.5% (n = 608) in Lobamba Lomdzala (Luyengo Clinic). The project achieved higher levels of engagement with each successive event (Table 2). A total of 172 participants (~ 10%) took advantage of onsite health consultations during the Girl Champ events, including HIV testing.

**Table 2:** Girl Champ event attendance, health system registrations, and reach

Inkhundla catchment area (clinic name)	Ludzeludze (Zombodze)	Mafutseni (Mafutseni)	Lobamba Lomdzala (Luyengo)	All clinics
Date of activation	11/10/2018	11/17/2018	11/24/2018	
Previously registered	154	57	247	458
New registrations	416	487	361	1264
Total registered attendees	570	544	608	1722
AGYW (15–24 years of age) population estimates	3544	2318	2291	8153
Estimated reach (attendance/population)	16.08%	23.47%	26.54%	21.1%

Chi-square analyses found there were no statistically significant differences in the proportion of 42,145 healthcare visits that were attended by AGYW before and after the Girl Champ Events (*X*^2^ = 2.4, DF = 1, 16.8% vs 16.2%, p = 0.121). However, there was a statistically significant difference in the proportion of the 4,247 HIV testing and counselling (HTC) visits that were AGYW before and after the Girl Champ events (*X*^2^ = 17.7, DF = 1, 30.5% vs 36.8%, p < 0.0001) which held for both age groups (i.e., AGYW between 15–19 years of age (*X*^2^ = 10.9, DF = 1, 10.0% vs 13.3%, p = 0.001), and 20–24 years of age (X^2^ = 5.2, DF = 1, 20.5% vs. 23.5%, p = 0.023).

This increase in the proportion of HCT visits accessed by AGYW was consistent with the perspectives of clinic staff we interviewed. They felt that the clinic-based Girl Champ events contributed to AGYW’s increased comfort with and knowledge of available services.

*I think from that event, we see a number of them coming in because they were introduced to staff, and they were able to see us, so now it’s easy for them to come because they know us. They also relate their issues freely…Now they know the different types of services that we offer. They are able to come to access them.* Regional Implementing Partner Public Sector

### Mechanisms Underlying Factors Associated with Girl Champ Outcomes

A total of 550 participant feedback surveys were collected across the three pilot sites. Participants from Zombodze made up 43% of evaluation responses (n = 240), followed by 31% from Mafutseni (n = 169), and 26% from Luyengo (n = 141). Participants ranged in age from 10 to 27 years old with a mean age of 16.7 years old. The majority of participants surveyed (58%, n = 311) were between the ages of 15–19 years old. Feedback from implementation stakeholders (interviews) and AGYW participants (surveys) offers insights into the facilitators and barriers to implementing the Girl Champ pilot (Table 3).

**Table 3:** Overview of key qualitative themes and descriptions

Facilitators	
Collaborative planning and mobilization: Girl Champ engaged both the health system and community members to drive high attendance at Girl Champ events, which was believed to be more effective than only engaging at one level	
Cohesion and shared identity: The Girl Champ brand was viewed as successful in terms of brand cohesion, innovation, appropriateness, and addressing sexual and reproductive health holistically for AGYW	
Barriers	
Logistical challenges in pilot implementation: Higher than expected attendance at events contributed to logistical challenges at events	
Sustainability of Girl Champ: Stakeholders expressed concerns about the sustainability of the brand, and continuing engagement with AGYW	

### Facilitators of Girl Champ Implementation and Effectiveness

#### Collaborative Planning and Mobilization

Key stakeholders described successful cross-sectoral working relationships as a major facilitator of Girl Champ success. A technical working group made up of representatives from the MoH (including the HPU, SNAP, and NERCHA) and facilitated by PLM was established at the onset of the project. This working group provided an effective forum for communication and participatory governance at the national level, with representation extending to the regional and local stakeholders as the pilot was planned and implemented in Manzini region. Participants noted how the project united different sectors and disciplines into “one team.” Stakeholders also appreciated the ways in which the working group facilitated strong planning and mobilization in support of the pilot.

*Really, we’ve learned from Project Last Mile…that the juice of success is proper planning. Know your teammate. Then, you’ll play your part.* Regional Implementing Partner

Participants also highlighted the importance of PLM’s work with clinics and their Health Committees to advertise the Girl Champ events and obtain buy-in for the brand from local communities. Further, participants felt that the integration of the Girl Champ communication strategy into existing community structures, including schools and community leadership, would help ensure the sustainability of Girl Champ.

*Cascading down to the health facilities, the health promotion unit, and engaging the community leadership was one of the strengths of reaching out to more people. During the activation, the adolescents and young women who had participated had authority from their parents and had authority from their caregivers to actually go and participate...* Regional Implementing Partner

*The strong participation and involvement of the clinic committee - which is a committee that’s selected by the community - made it so much a community-owned process. If we invest in that, the likeliness that it’s sustainable - and people understand it - is higher. The strength of PLM is building it around the clinic.* Public Sector Representative

Stakeholders felt that the strong community mobilization and buy-in were the key drivers of the higher than anticipated attendance at the Girl Champ events. The word-of-mouth mobilization was viewed as far more valuable than the use of traditional media (such as radio) in promoting the brand and events.

*Mobilization of the girls to come was largely done by teachers and part of the community. When it was compared to what radio did, we saw that there was more coming from the community…* Public Sector Representative

*It wasn’t radio. It wasn’t social media. It was the community engagement where [PLM] went into the communities and spoke to the community leaders, and they distributed the information. A lot of the schools also brought in a lot of the kids. Teachers would put in the information that they required and brought them in. I think the word of mouth was the strongest component.* Project Last Mile Representative

The participant feedback forms further reinforce the importance of community mobilization in recruitment and attendance at events. A total of 133 participants from two sites reported where they heard about the event, with the majority learning about Girl Champ through schools (58.6%), friends (11.3%), or family (10.5%) with a further 9.8% hearing either from the media or health care workers, respectively.

#### Girl Champ was a Cohesive Brand, Offering a Shared Identity for AGYW

Participants described Girl Champ as a cohesive, innovative, and cross-cutting communications strategy. The brand was not linked to a specific clinic, partner, or health need; rather, the brand promoted overall health and wellness and sought to speak directly to the identities and aspirations of AGYW. This made Girl Champ immediately appealing to AGYW, and also offered the potential for Girl Champ to be used across diverse health programming for issues beyond HIV.

*What’s also crucial that the brand is just the Girl Champ. And there aren’t partners logo[s] so much in that material. If you put a national logo, for instance, you repel the audience because they already know what is going to come out of that. If ICAP appears, if whosoever appears in the logo, they already know it’s HIV. Research has also shown us that people are quite fatigued. We need to be innovative about that…It looks like an independent brand and resonates with the people, then when they get there, they get surprised by the content, that it’s still information and services on HIV and trying to get them to services. I think that’s also a great success.* Public Sector Representative

Stakeholders also appreciated how the branding and merchandise incorporated local design elements, contributing to a sense of identity with the brand, particularly among the target audience of young women who attended the Girl Champ events. One stakeholder described how the merchandise and safe space at events fostered this identity, creating a *girl squad…all united in their girl championess.* The Girl Champ brand supported this unified sentiment even after events, as stakeholders described young women coming to clinics identifying themselves as Girl Champs.

*That idea, I think it was a great one because the girls felt like they have an identity. They own something because when they come, you’ll even hear them outside that “I’m a Girl Champ” because most of the time, when they come to the clinic, they come wearing their t-shirts. You can find that they feel like there is an identity for them.* Regional Implementing Partner, Public Sector

To a slightly lesser extent, stakeholders also felt that the Coach concept provided a fresh and cohesive identity for nurses and clinic staff that promoted more confidential, objective, and youth-friendly care.

*I think the Coach curriculum was beneficial to sort of brush, or maybe really polish the soft skills for delivering of services. Especially the part of being objective and being a professional even though nurses are mothers, nurses are grandmothers…I think it was reinforcement of the adolescent and youth services delivery model.* Regional Implementing Partner

Both the interviews and the participant feedback forms indicated that Girl Champ delivered on the brand promise of making AGYW feel supported and respected in a girls-only safe space during events that were fun and youth-friendly. Feedback from AGYW on the Girl Champ event was positive, as 95.5% of participants indicated they enjoyed the experience, 99.2% would recommend the experience to friends, 96.2% would share knowledge that they learned, and 93.5% indicated that the healthcare workers at the event made them feel comfortable. AGYW sought more information on puberty, family planning, and sexual and reproductive health in future events.

AGYW described what they liked and did not like about the event in open text fields where participants could write-in multiple responses, which were coded for descriptive analysis. Roughly 45% of the responses indicated that what they liked most was the Q&A with healthcare workers, the health information they received, and the safe space that enabled them to dialogue at the event. This is particularly encouraging since these are sustainable intervention components that align with current health promotion activities in primary care facilities. As one respondent noted in her feedback, *I enjoyed being me. I'm happy that I spoke to other girls and they listened to my problem. I'm so relieved that I spoke about what I wanted to know and what I needed answers to.* A further 31% and 20% of respondents indicated they enjoyed the entertainment and fitness activities the most, respectively. The giveaways, food and onsite health services featured less prominently in the feedback forms.

Stakeholders also described the value of the activities provided during the event. One senior health official even encouraged his own daughters to participate after he attended a Girl Champ event. Young women’s openness and active participation in the Q&A was described by stakeholders as evidence of emerging trust between young women and health care providers.

*…we’re amazed how outspoken the girls were. They were talking through issues of their concern. You could tell that there’s a gap in terms of knowledge of the health issues and, again, we’ve never limited ourselves to HIV. We spoke across the board on health issues from growing up, nutrition, engaging in sexual activity, and what other factors that contribute to HIV, contracting HIV. [The girls] were very open. We had to adjust. Some of us had to adapt. We needed to be at their level and very professional…They were asking very pertinent questions that were related to their health.* Public Sector Representative

*…the young women and girls still see health workers as a preferred source of information. They still see a health facility as the preferred source of where they can go for help. We sometimes have community activities in the health facilities, but it’s the normal boring kind of services. At the activation…the young people were at home…it demystified the health facility for some who were real scared to go there.* Public Sector Representative

Health care providers in particular felt that this demystification of the clinic space, combined with opportunities for positive interactions with nurses and staff, were key facilitators of AGYW’s health-seeking behavior after the Girl Champ activation events.

### Barriers to Girl Champ Implementation and Effectiveness

#### Logistical Challenges

The primary challenges described by stakeholders were logistical issues occurring during the Girl Champ events. At all three Girl Champ events, the numbers of participants far exceeded the original estimate (1722 actual vs. 750 targeted) straining available space and resources for the events. One event had to be moved from the clinic site due to limited space. PLM and the Girl Champ team also faced shortages of food and branded materials needed for the large number of attendees. Further, the volume of participants overwhelmed the available local transportation, resulting in delays in AGYW getting to and from the events, with some participants waiting up to 2 h.

*On the first activation… we had 250 girls arrive, and then we had 500 girls arrive, and then we had almost 750 girls arrive…We didn’t expect to have such inflow...If there is a party or something going on, people wanna come…we had said from the beginning that we wouldn’t turn anyone away, so once you were at the door, you had to come in. This now leads up to the cost implications afterwards, but obviously we had to use merchandise that was bartered through the next activation at this activation.* Private Sector Representative

AGYW’s feedback on what they did not like also primarily centered on logistics with transport issues, lavatories, and a handful of respondents who did not receive food and/or giveaways that seemed to stem from the greater-than-anticipated attendance at all three events.

#### Sustainability of Girl Champ

Stakeholders expressed urgency to refine and expand the Girl Champ strategy to ensure long-term engagement with AGYW, and shared concerns about the sustainability of Girl Champ with available resources. Specifically, participants identified the need for additional events stratified by age, and the need to create resources such as toolkits that would allow others in the eSwatini health system to adopt the strategic marketing process and strategy.

*I think now that we’ve got these girls, and they are good to go and were activated—but then what’s this last thing, this sustainability plan around getting these girls? Why were we getting them if we are not following up? Maybe having girls’ clubs or whatsoever that are following the Girls Champs system or type of intervention…They were asking questions, and they were happy that we were answering questions, and they were getting to have this relationship with us as healthcare workers. But then after the activation, we are gone.* Public Sector Representative

Additional events and resources would allow continued engagement with AGYW, ensure that future health communication approaches are similarly embedded, and build capacity to enable replication of the strategic marketing process with other populations and initiatives. Participants were especially concerned about maintaining momentum:

*How do we guide the people that we can’t activate… in terms of giving them the tools? How do we educate everyone, but without doing all events and having a guideline on that? It’s about cementing the movement within the country more and having a way where the girls can engage with us a little bit more.* Private Sector Representative

## Discussion

The Girl Champ brand in eSwatini combined strategic marketing expertise and best practices from The Coca-Cola system, with locally driven community mobilization through existing public sector structures and health provider sensitization to improve demand for HIV services among AGYW in eSwatini, a segment of the population for whom engagement in prevention and treatment is deeply lagging. Our mixed-methods evaluation of the pilot in three municipalities showed that the approach was well received (with event attendance far outstripping projections and participating AGYW providing overwhelmingly positive feedback) and was associated with longitudinal improvements in demand for HIV counselling and testing services among AGYW in the two clinics for which electronic administrative records were available. Despite the increase in the proportion of HIV testing and counselling visits, there was not significant change in the proportion of overall healthcare visits attended by AGYW. This may be because the intervention impacted only the most targeted behavior (HIV testing) and was not generalized to demand for other services, or it may be because the sample size from two clinics was insufficiently powered to detect the more generalized change. Further longitudinal review of overall attendance and visit types at comparison clinics in the region is underway by this authorship team. Nonetheless, Girl Champ is consistent with other person-centered and gain-framed efforts to successfully engage AGYW in HIV services [55–57].

We also identified factors that facilitated the successful implementation of the Girl Champ pilot and contributed to the effectiveness of Girl Champ in engaging AGYW. Collaborative planning and community mobilization through schools and community leadership were critical to the higher than anticipated attendance at Girl Champ events. The value of engaging community leadership and participatory intervention development is well-known in promoting HIV and sexual health services, and Girl Champ illustrated the critical role such leadership can play in disseminating health communication campaigns to adolescents [58–60]. In particular, schools play an important role in youth lives and sexual and reproductive health, as a venue for accessing services, information, and support [61–68].

At the same time, integrating HIV prevention into community structures and cultural practices can be complex [69, 70]. Gender-segregated events for youth, such as the *Umhlanga* (Reed Dance) for young women, are an important part of eSwatini coming-of-age and discussions of youth sexuality, but these rituals are also viewed as a potential source of sexual risk and gender inequity [69]. The “girls-only” nature of Girl Champ mirrors some aspects of *Umhlanga* and resonated with both participants and key stakeholders. However, we lack the data to say whether parallels between *Umhlanga* and Girl Champ events played a role in implementation or how stakeholders or young women perceived the events in comparison. Future work could investigate the complex nature of this practice with respect to HIV social marketing interventions, and whether Girl Champ draws on this practice implicitly as a cultural reference.

Another key facilitator of the Girl Champ pilot was the brand itself, which built on aspirational or gain-framed approaches to adolescent sexual and reproductive health that have been recently recommended in the literature [12, 32, 40]. Such messaging may be particularly effective when interacting with youth who perceive themselves as less vulnerable to negative health outcomes [71, 72]. Participants also noted the importance of Girl Champ as a strong standalone or partner-agnostic brand, without branding associated with government or NGO partners. This is consistent with strategic marketing principles and campaigns that have targeted other health behaviors, with practical implications for others seeking to employ similar approaches [73, 74]. The integration of eSwatini’s electronic health record system into Girl Champ events was perceived by stakeholders to be another novel element of Girl Champ that could promote follow-up of AGYW in the future. Given increased interest in assessing and improving adolescent engagement in HIV testing and care in the “test and treat” era, linking adolescents to electronic health systems could be a critical component for similar demand creation interventions [75].

Sustainability and scale-up of Girl Champ were identified as key concerns. Girl Champ was intended to be a pilot effort at shifting demand for HIV testing and care in a relatively short period of time. As such, additional work is needed to identify the critical components of the Girl Champ intervention that could be scaled-up in other parts of eSwatini and sustained or amplified to engage AGYW in healthcare post-COVID. The concerns about Girl Champ’s sustainability and long-term impact are consistent with literature suggesting that there is a need to build capacity for strategic marketing in public health and more rigorously evaluate such interventions at scale [38].

Our study should be interpreted in light of its limitations. First, the quantitative CMIS data on health service uptake is limited to two clinics, as one of the clinics was not on the CMIS. While the missing clinic’s data presents a limitation, this study was a pilot that was not intended to be fully powered to assess impact at the individual clinic level. Furthermore, one of the clinics out of the two with available data had relatively smaller caseloads. Further study is needed to assess clinic- and community-level factors that would mitigate the effectiveness of such interventions, including clinic size. Second, qualitative data may be subject to social desirability bias, as stakeholders may seek to represent the pilot as a success. However, we identified common themes across participants, sought detailed descriptions of the process of planning for and implementing the pilot, and probed for negative experiences and recommendations [76]. Third, the relatively small qualitative sample size may limit transferability to other settings. Despite the small sample size, our findings identified themes that resonate with the literature and offer important lessons concerning the implementation of a strategic marketing intervention.

## Conclusions

Our evaluation of the Girl Champ pilot found that an integrated intervention that includes strategic marketing, community mobilization, and health provider sensitization offers a promising approach to improve adolescent demand for HIV testing and counselling services. Lessons learned from the Girl Champ pilot can inform scale-up of Girl Champ in eSwatini, as well as guide others seeking to incorporate private sector marketing approaches into HIV prevention interventions for AGYW in other low- and middle-income settings.

## Supplementary Information

Below is the link to the electronic supplementary material.

Supplementary file1 (DOCX 26 KB)

## Data Availability

The qualitative interview guide and final codebook are available as appendices.
