# Supplementary material for: Girl Champ in eSwatini: A Strategic Marketing Campaign to Promote Demand for Sexual and Reproductive Health Services Among Young Women
Source: AIDS Behav. 2021 Aug 31;26(3):853–63. doi: 10.1007/s10461-021-03446-y (PMC8840893; doi:10.1007/s10461-021-03446-y)
Supplement: Supplementary file 1 — Supplementary file1 (DOCX 26 KB) [file 10461_2021_3446_MOESM1_ESM.docx]

**Supplemental Materials**

This appendix includes the interview guide for the qualitative stakeholder interviews, and the qualitative codebook.

**Project Last Mile Swaziland {eSwatini}**

**Semi-Structured Interview Guide**

**Purpose:** The interviews to be conducted in March 2019 are being held to understand implementation of Project Last Mile in eSwatini [Swaziland], as well as highlight the strengths and challenges that have influenced the work at hand. Our goal is to understand lessons learned in eSwatini [Swaziland] and to share these with other similar projects that may take root in Africa. We are interested in learning about possible shortcomings of Project Last Mile, opportunities for improvement, and success stories.

As you know, PLM is a partnership to bring private sector expertise from the Coca-Cola Company to the public health sector. This was the first demand creation project for the partnership which planned to target young women and girls for health services, entitled Girl Champ. The interview questions are open-ended and invite you to reflect on your experience with the project since Girl Champ was proposed and launched in November 2018.

*We would like your permission to record this interview. This lets us listen carefully to you rather than taking notes and ensures that we will accurately capture our conversation. All information will be kept strictly confidential and no identifying information about you or your organization is included on the transcript. Digital files with audio-recorded material will be deleted as soon as the transcripts have been reviewed for accuracy. If at any point you would like me to turn off the recorder, please let me know. You are free to decline to participate, to end our interview at any time for any reason, or to choose to skip any question.*

**Discussion Points:**

1. Please describe your role in your organization.
   1. How long have you worked in this position?
   2. And what is your role with Project Last Mile?
2. How has the project been going in the past year?
   1. What has been going well?
      1. Can you provide some examples of these strengths or successes? *(probe for concrete examples and for details on each)*
   2. What are some things that have not gone so well? Any setbacks, or challenges so far?
      1. How were you able to address these challenges? *(probe for concrete examples; especially how they have addressed the challenges)*
   3. Has the project been different than you expected and if so can you talk about that?
3. Have there been any changes in the way demand creation or health promotion have worked since Project Last Mile got involved? If so, how? How do you think these changes will be sustained?
4. How would you describe the working relationships you have developed through PLM? *(probe for concrete examples)*
5. How has the communication between the team and different stakeholders been going? What has worked and what hasn’t?
6. Is there anything I haven’t asked you that would be helpful for us in understanding PLM in Swaziland [eSwatini]?

**NOTE:** At each step along the interview, the interviewer should **follow the story and ask questions to help us understand the story.**

For instance, when asked about the engagement, the respondent might say *"We put a working group together"* to which the interviewer might ask *"Who put the team together? Who was on it? Why were these people included? What were your hopes for what the working group would achieve?”*

**General probes:**

- Can you tell me more about that?
- Help me understand what you meant by “___________?

**Content Probes:**

**Key project activities**

Stakeholder Engagement & Communication: There are many government, NGO, academic, and donor partners engaged in Swaziland [eSwatini] in the health promotion space.

- How would you describe the stakeholder relationships that have developed through PLM?
- How has the communication structure between PLM and the stakeholders been going?
  - How is the Working Group functioning? Are they able to meet regularly and efficiently?
  - How receptive was the District to the activities that were being proposed? HCWs? Others?

Preparing the Landscape for Implementation: Preparation was needed before Girl Champ could be piloted in Manzini district.

- How would you describe efforts to engage the district partners and health facilities in the strategy?
- How were these efforts received by health care workers and front line managers?
- How did communities react to mobilization activities? If successful, what contributed to their success? If they did not go well, what were the challenges?
- How successful were other aspects of the media strategy – radio, billboards, social media? What worked and what didn’t?

Demand Creation and Organization Capacity:

- For HCWs: How was the COACH training? How was the refresher training conducted by ICAP? What worked and what didn’t work in terms of building capacity?
- For District/HPU/SNAP/NERCHA: How was the training in preparation for Girl Champ by FCB? What worked and what didn’t work in terms of building capacity?
- How sustainable do you think these learnings were? Do you think people who were trained will be able to continue to use those skills? [i.e., will HPU and NERCHA be able to translate these skills for strategic marketing to other health concerns/target populations? If yes, how? And will the HCWs be able to remain COACHes? If yes, how?
- How do you think the demand creation and health promotion capabilities will change -if at all- after Project Last Mile? How would these efforts be sustained? What is needed to drive and continue this change?

Girl Champ Brand and Events

- What did you think the Girl Champ brand and materials? What did you like and not like?
- What did you think of the Girl Champ events and activities? What did you like and not like?
- What can be done in future to improve the brand, and create demand in future?

**Key quantitative metrics (impact indicators)**

- What impact do you think this work will have on young women and girls in eSwatini? On youth in general? On the Manzini community?
  - Improved uptake of health services – eg., HIV testing, SRH/Family Planning, PREP, STI Screening
  - Improved self-esteem, power to negotiate safe sex/ ‘stop and think’, navigate clinic, clinic
  - Improved wellness – fitness, self-care, nutrition
  - Improved social support – safe space to dialogue, seek expert advice, speak up
  - Improved health service delivery for AGYW – COACH training, compassionate, confidential, objective care.

**PLM Code Book**

Note: Do not use any codes with a “retired” prefix.

| **Code Name** | **Definition** |
| --- | --- |
| **100. Context** |  |
| 101. Context | Enabling/disabling regulatory, legal and political factors; policy landscape; country context and culture; politics |
| **200. PLM Process** |  |
| 201. Knowledge transfer | Between Coke/PLM and other partners, sharing expertise, capacity building; actual transfer of knowledge or capabilities. Excludes private sector inputs |
| 202. Private sector inputs_Technical | Technical expertise that PLM/Coke is providing to public sector partners, such as tools, data, analytic strategies, soft inputs, etc. Excludes marketing and communication inputs |
| 202.a. Private sector inputs_Marketing&Comm | Marketing and communication inputs being provided by PLM/Coke to public sector partners. Includes assistance with branding, franchising, market research, creative design, demand creation, etc. |
| 202.b. Private sector inputs_Expectations | Expectations about what the private sector/PLM/ Coke will bring in terms of technical inputs, training, innovations, marketing, capacity building, tools, software, etc. that may or may not be met. |
| 203. Adaptation/flexibility | Adaptations/shifts in response to circumstances, examples of how something was adapted to fit circumstances, context |
| 204. Networks | Boundary spanners, active use of pre-existing relationships to make connections, development and dynamics of relationships that contribute to the work |
| 205. Assessing and aligning “fit” between PLM and external partners | Assessment of the landscape, and planning for how PLM is/will be involved; Coordinating and finding complementary roles, complementary expertise; addressing role conflict/overlap and competing demands/priorities amongst partners. |
| 206. Process | Description of processes through which PLM contributes, the tasks and work done by PLM. The steps PLM has followed to reach their current involvement. Might include knowledge transfer, but not necessarily. |
| 207. Data sources and evaluations | Any metrics or data that has been used in evaluating different components of PLM |
| 208. Historical explanations | Explanations for how partnerships, processes, other aspects came to be. |
| 209. Impact/sustainability | References to long-term results or impact of PLM project, including sustainability of the project after PLM implementation. Could include discussion of factors that may influence long-term results and sustainability. |
| 210. Boundary spanner | Refers to a particular person in the PLM partnership who is able to communicate across public and private sectors |
| 211. Governance | Refers to PLM project oversight, roles and responsibilities, planning and deadlines; also can refer to how PLM is governed by external structures and situated within organization |
| 212. Route Optimization | Refers to PLM workstreams related to optimizing routes for distribution, including geomapping, supply chain logistics, route delivery models, last mile logistics, etc. |
| 213. Capacity Building | Refers to PLM workstreams related to specific capacity building, such developing organization structures, job descriptions, and performance management processes for the public sector |
| 214. Outsourced Distribution | Refers to PLM workstreams related to facilitating and negotiating outsourced distribution |
| 215. Demand Creation | Refers to PLM workstreams related to facilitating demand creation, including marketing research, creative agency development, strategic communications, organization development, messaging |
| 216. Innovation | Refers to innovation or lack thereof; when a respondent refers to something as a new way of doing things or explicit reference to an innovation that was contributed by PLM. Can also refer to expectations for innovation and whether the partnership met that expectation (and how). |
| 217. Branded Service Organization | Refers to PLM workstreams that relate to rebranding organizations or services with a new brand/ identity, look and/feel; core values; logo development; service orientation. |
| 218. Visibility and Analytics Network (VAN) | VAN is an acronym for “Visibility and Analytics Network.” VAN encourages a more holistic approach to supply chain improvements. By implementing software tools that provide access to crucial data, defining indicators and clear management actions based on the values for those indicators, as well as creating a cadre of professionals responsible for improving the supply chain. VAN represents a new, data-driven approach to supply chain improvement, and I am excited to play a role, helping to drive this innovation forward. |
| **300. Influences on PLM process** |  |
| 301. Inter-sectoral communication | Communication across organizations/partners, such as between ministries of health and private sector partners. Excluded: communication within a single unit (department, level of system, or organization). |
| 302. Communication within units | Communication within a department, level of the system, or organization. |
| 303. Organizational culture | Descriptions of learning and problem-solving, ways of working together, commitment to organization, team work. |
| 304. Buy-in and ownership | Extent to which individuals at different levels of the system feel ownership and want to be a part of PLM or PLM-supported programs, part of team |
| 305. Trust and motivation for partnerships | Feelings of trust (or lack thereof) between partners, in the development of relationships and partnerships. Also can indicate alignment on good will, public benefit or lack thereof (i.e., potential for conflict of interest). |
| 306. Momentum | References to the pace at which PLM’s work has moved; ebbs and flows in progress. |
| 307. Funding/Resources | Refers to availability of financial/monetary resources as well as human resources. |
| 308. External communications and advocacy | Refers to communication about and dissemination of PLM’s work to organizations and stakeholders outside of the partnership |
| 309. Bureaucracy and Contractual Considerations | Refers to contractual issues, bureaucratic processes, ‘red tape’, regulatory requirements and how they affect the partnership |
| 310. Logistics | Refers to logistics and how they affect the partnership – e.g., vehicle breakdowns, per diem issues, meals, venue hurdles, transportation [not related to logistics capability workstream] |
| **900. Overarching codes** |  |
| 901. Change | Any changes as a result of PLM’s involvement; Changes to CCMDD; To be applied in addition to whatever changed (communication, organizational culture, etc.) |
| 902. Barriers | Anything that has inhibited PLM, partnerships, CCMDD. To be applied in addition to whatever the barrier was (communication, organizational culture, etc.) |
| 903. Facilitators | Anything that has facilitated PLM, partnerships, CCMDD, helped in overcoming challenges. To be applied in addition to whatever the facilitator was (communication, organizational culture, etc.) |
| 904. Great quote | Quotations that are particularly illustrative of particular themes. |
| 905. Successes | Anything described as a successful outcome of PLM. May or may not also be coded as change. |
| 906. Feedback and Recommendations | Information that might be useful to provide as feedback to partners (delivery team, PMO, steering/working committees). Could be double-coded with a code that is specific to the recommendation, but not necessary. |
